# Fabrication and Biocompatibility Evaluation of Nanodiamonds-Gelatin Electrospun Materials Designed for Prospective Tissue Regeneration Applications

**DOI:** 10.3390/ma12182933

**Published:** 2019-09-11

**Authors:** Aida Şelaru, Diana-Maria Drăgușin, Elena Olăreț, Andrada Serafim, Doris Steinmüller-Nethl, Eugeniu Vasile, Horia Iovu, Izabela-Cristina Stancu, Marieta Costache, Sorina Dinescu

**Affiliations:** 1 Department of Biochemistry and Molecular Biology, University of Bucharest, 050095 Bucharest, Romania; aida.selaru@bio.unibuc.ro (A.Ş.); sorina.dinescu@bio.unibuc.ro (S.D.); 2 Advanced Polymer Materials Group, University Politehnica of Bucharest, 011061 Bucharest, Romania; diana.dragusin@upb.ro (D.-M.D.); elena.olaret@stud.fim.upb.ro (E.O.); andrada.serafim@gmail.com (A.S.); horia.iovu@upb.ro (H.I.); izabela.stancu@upb.ro (I.-C.S.); 3 DiaCoating. GmbH, 6112 Wattens, Austria; doris.steinmueller@diacoating.com; 4 Department of Science and Engineering of Oxide Materials and Nanomaterials, University Politehnica of Bucharest, 011061 Bucharest, Romania; eugeniu.vasile@upb.ro; 5 Research Institute of University of Bucharest, 050107 Bucharest, Romania

**Keywords:** tissue engineering, diamond nanoparticles, fish gelatin, adipose-derived stem cells, biocompatibility

## Abstract

Due to the reduced ability of most harmed tissues to self-regenerate, new strategies are being developed in order to promote self-repair assisted or not by biomaterials, among these tissue engineering (TE). Human adipose-derived mesenchymal stem cells (hASCs) currently represent a promising tool for tissue reconstruction, due to their low immunogenicity, high differentiation potential to multiple cell types and easy harvesting. Gelatin is a natural biocompatible polymer used for regenerative applications, while nanodiamond particles (NDs) are used as reinforcing nanomaterial that might modulate cell behavior, namely cell adhesion, viability, and proliferation. The development of electrospun microfibers loaded with NDs is expected to allow nanomechanical sensing due to local modifications of both nanostructure and stiffness. Two aqueous suspensions with 0.5 and 1% w/v NDs in gelatin from cold water fish skin (FG) were used to generate electrospun meshes. Advanced morpho- and micro-structural characterization revealed homogeneous microfibers. Nanoindentation tests confirmed the reinforcing effect of NDs. Biocompatibility assays showed an increased viability and proliferation profile of hASCs in contact with FG_NDs, correlated with very low cytotoxic effects of the materials. Moreover, hASCs developed an elongated cytoskeleton, suggesting that NDs addition to FG materials encouraged cell adhesion. This study showed the FG_NDs fibrous scaffolds potential for advanced TE applications.

## 1. Introduction

The field of regenerative medicine and tissue engineering (TE) has emerged as a new approach for serious trauma injuries to essential organs of the body. Due to the considering disadvantages, namely insufficient donors, incompatibility between donors, and immune system issues that come along tissue grafting and organ transplants, TE holds a great promise towards regenerating the injured organ and, more importantly, gaining back the essential body functions towards normal life. Although serious steps are made in the field of microsurgical interventions, these still do not deliver the expected result in the setting of organ trauma [1]. Therefore, TE as a new and modern biological approach is being developed in order to overcome these challenges. TE combines the principles of engineering and life science in order to generate biological substitutes that will assist the injured tissue in regenerating and achieving normal functions. The key players in the frame of TE are cells, scaffolds and growth factors, molecules or nanoparticles which serve to stimulate a better interaction between the cells and the scaffold [2].

Since this field emerged, substantial efforts have been made to generate natural based polymers for biomedical applications, drug delivery, and regenerative medicine. In comparison to synthetic polymers, natural ones generate lower immune responses and better interact with the cellular components in terms of viability and toxicity [3]. Among them, fish gelatin (FG) is becoming one of the most promising biopolymer not only for medical applications, but also widely used for cosmetic and pharmaceutical purposes [4], due to a low risk of transmitting diseases as in case of mammalian gelatins [5]. Gelatin is a collagen derivate, obtained by partial hydrolysis of native collagen, therefore becoming attractive for scaffold preparation as it can deliver a similar structure to the extracellular matrix (ECM) found in most animal and human tissues [6,7], that can ensure appropriate chemical and biological cues for strong interactions with a large variety of cells [8]. In the past, several studies demonstrated that gelatin-based scaffolds are bio-friendly and interact well with different cell types, such as fibroblast [6,9], murine pre-osteoblasts [10], rat corneal keratocytes [11], mature adipocytes [12] and neonatal mouse cerebellum stem cells [13]. It has a wide use in the field of TE also due to its capability to deliver growth factors and to enhance the vascularization process within newly engineered tissue [14]. Thus, this polypeptide plays a key role in designing functional scaffolds, providing finely tuned templates which can facilitate cell adhesion, growth, proliferation, and differentiation. Therefore, gelatin has a great potential to serve as a substrate for multiple TE applications, including bone, adipose, corneal and nervous tissue regeneration.

Modern trends in scaffolding techniques include the functionalization and reinforcement at nano and micro scale of materials with different types of nanoparticles, such as gold nanoparticles, carbon nanotubes (CNT), halloysite nanotubes (HNT), diamond-like carbon (DLC) and diamond nanoparticles (NDs) [15], since cells seem to sense and respond to nanoscale or microscale in terms of adherence, growth, and proliferation [16]. Diamond, a material composed of carbon atoms arranged in a cubic crystal structure, has become a potential candidate for biotechnological and life sciences applications, especially due to its natural provenance. Mostly, it became attractive for a large range of TE applications under its nanostructured form, specifically NDs [17]. Moreover, NDs turned out to exhibit low or no cytotoxicity when brought in contact with cells [18]. This type of nanoparticles has been mostly explored for hard tissue regeneration, such as bone [19,20,21], yet carbon-based structures’ potential for supporting regeneration is also evaluated for softer tissues, such as the skin [15,22] or the nervous tissue [23].

Stem cells are widely used as a tool in regenerative medicine and TE, due to their proliferation abilities and high potential to differentiate to multiple cell types. While embryonic stem cells (ESCs) and induced pluripotent stem cells (iPSCs) can follow multiple cell lineages belonging to all three embryonic sheets, adult stem cells maintain their versatility, but with a more reduced ability of differentiation. Among the adult stem cells types, adipose-derived mesenchymal stem cells (ASCs) can be easily isolated from fatty tissue due to minimal invasive procedures, such as liposuction. ASCs are able to undergo differentiation towards adipogenic [24], osteogenic [25] and chondrogenic [26] lineages. Interestingly, ASCs seem to be capable of differentiating also to several cell types originating from the ectoderm [27]. In this respect, very recent work demonstrated that these kind of cells are able to form neurospheres after 6 days of culture in specific culture media, being positive for microtubule-associated protein 2 (MAP2), glial fibrillary acidic protein (GFAP) and sex-determining region Y-box 2 (SOX2) neural markers [28]. All these characteristics proved that ASCs deliver expected behavior in engineered scaffolds and could be the solution for clinical applications.

It has been reported that the performance of stem cells behavior could be controlled by the presence of a nanostructure within the scaffold [29]. It seems that once cells interact with the nano-components, their adhesion, growth, and migration is modulated. Reinforcing natural based materials with these structures is a modern strategy for stem cell guidance and brings a great contribution to the vast field of TE [30]. The premise of our study is that cells respond differently to mechanical stimuli. As it was shown in previous studies, when in contact with an implantable device or scaffold material, the cellular behavior is influenced by the nanoscale or submicroscale characteristics of the surface [31]. In their study, Darling et al. [32] have shown a close connection between the cell morphology and the young modulus of the surface with which they interacted. Thus, hASCs exhibited a spread morphology when in contact with a stiffer surface than when in contact with a more elastic surface, the second substrate leading to a spherical cell morphology.

In this study, we sought to better understand the effect of the NDs on the nanomechanical properties of fibrous gelatin-based nanocomposite hydrogels and to investigate the mechanisms of the physical factors that could influence the cell response regarding adhesion, proliferation and cell morphology. Therefore, we expected that the incorporation of the nanoparticles within the polypeptide fibrous structure to contribute to the guidance of hASCs through mechanical sensing, leading to enhanced cellular activity. In this context, the aim of our study was to fabricate and characterize electrospun fibrous gelatin nanocomposite meshes containing NDs in terms of mechano-structural properties and the ability to support hASCs adhesion, growth, and proliferation, as a potential platform for tissue regeneration.

## 2. Materials and Methods

### 2.1. Preparation of Electrospun NDs-loaded FG Fibrous Scaffolds

Surface functionalized (COOH and OH) NDs suspension in ultrapure water was kindly provided by DiaCoating (Wattens, Austria). The precursors for the fibrous nanocomposite scaffolds were obtained by dissolution of FG (Sigma-Aldrich Co, Oakville, ON, Canada) into double distilled water (ddw) or NDs suspensions until a final protein concentration of 50% w/v and various ratios of NDs (0%, 0.5% and 1% w/v). The gelatin was left to dissolve at 40 °C under vigorous magnetic stirring for 4 h. For a homogenous distribution of the NDs all the mixtures were sonicated in an ultrasound bath (Elmasonic S 30/H, Elma Schmidbauer GmbH, Singen, Germany), for additional 4 h.

To fabricate the electrospun fibrous scaffolds (further denoted FG, FG_NDs 0.5% and FG_NDs 1%, respectively), climate-controlled electrospinning equipment was used (EC-CLI, IME Medical Electrospinning, Waalre, The Netherlands). Accordingly, each solution was loaded in a 5 mL syringe and placed on a syringe pump to precisely controlled flow rate. A constant volume of 350 µl of each precursor was injected through a G22 nozzle, using a flow rate of 12.5 µl min^−1^ at a constant temperature of 25 °C and a relative humidity of 45%. Randomly oriented fibers were collected on a cylindrical collector (Ø 20 mm, rotating at 100 rpm), at a distance between the tip of the nozzle and the collector of 12 cm and a speed of the nozzle lengthways of the collector of 5 mm s^−1^ (to ensure a homogenous deposition of the fibers on the entire length of the collector). The applied voltage depended on the composition of the injected precursors ranging from 21 kV for FG and FG_NDs 0.5% to 23 kV for FG_NDs 1%. Cross-linking was performed on fibers detached from the collector, using an 0.5% w/v ethanolic glutaraldehyde (GA) solution for 4 days, at RT (GA, 50% w/v aqueous solution, Sigma-Aldrich Co, St. Louis, MO, USA). Forwards, the crosslinked fibrous scaffolds were extensively washed with ethanol (analytical grade, Chimopar, Bucharest, Romania) for two days, and finally with double distilled water.

### 2.2. Characterization of the Fibrous Scaffolds

Rheological behavior of all precursors was assessed under isothermal conditions using a Kinexus Pro rheometer (Malvern Panalytical Ltd, Malvern, UK) with parallel plate geometry. The 20 mm diameter disposable plates were used and the gap between the plates was fixed at 1 mm. Steady state flow tests to measure the viscosity of sample formulations were performed at room temperature at different shear rates from 0.01 s^−1^ to 1000 s^−1^.

Attenuated total reflectance Fourier transform infrared (ATR-FTIR) spectrometry was performed using a Jasco 4200 spectrometer (JASCO Deutschland GmbH, Pfungstadt, Germany) equipped with a Specac Golden Gate ATR device (Specac Ltd, Orpington, UK), at a resolution of 4 cm^−1^, in the wavenumber region of 4000–600 cm^−1^ and recording 200 scans/sample.

The morpho-structural features of the gold-sputtered fibrous scaffolds were examined through scanning electron microscopy (SEM) and high-resolution electron microscopy (HRSEM) using a Quanta Inspect F SEM device equipped with a field emission gun (FEG) (Fei Company, Hillsboro, OR, USA) with 1.2 nm resolution and with an X-ray energy dispersive spectrometer (EDX) (Fei Company, Hillsboro, OR, USA). Transmission electron microscopy (TEM) was also used in order provided information regarding the distribution of the NDs. The micrographs were registered using a TECNAI F30 G2 STWIN microscope (Fei Company, Hillsboro, OR, USA) operated at 300 kV with EDX and EELS facilities. Thus, a small piece of fibrous scaffold was deposited on a TEM copper grid and covered with a thin amorphous carbon film with holes. Single fibers were observed at the edge of the scaffolds.

Micro-computed tomography (microCT) investigation has explored the overall architectures of both mesh and tubular structures obtained by rolling the mesh around a plastic support. A SkyScan 1272, high-resolution X-Ray microtomograph (Bruker MicroCT, Kontich, Belgium) has been used. The samples were fixed with dental wax and placed in the scanning chamber, and the analyses were performed using an accelerating voltage of 45 kV and a beam current of 200 µA, with no filter present during scanning. The rotation step was set at 0.3 degrees. Images were processed using NRecon (Version 1.7.1.6, Bruker MicroCT, Kontich, Belgium), CTVox (Version 3.3.0r1403, Bruker MicroCT, Kontich, Belgium) and Data Viewer (Version 1.5.4.6, Bruker MicroCT, Kontich, Belgium) softwares.

Substrate surface contact angle was evaluated with a Drop Shape Analyzer 100 (DSA 100, KRÜSS GmbH, Hamburg, Germany), equipped with a high-resolution camera for capturing images during the measurements and evaluated using ADVANCE software (Version 1.7, KRÜSS GmbH, Hamburg, Germany). To avoid user-related errors and to assure repeatability an automatic program was defined in the software for the dosing system. A volume of 2 µl of double distilled water was placed on each surface, at room temperature (25 °C). Measurements were registered for 3 s (10 fps) and the drop’s shape was analyzed using a sessile drop technique. For each sample, four measurements were performed on different areas and the values were averaged.

Mechanical properties at nanoscale (the Young’s modulus, (E) and hardness (H)) were investigated by nanoindentation using a Nano Indenter G200 (Keysight Technologies, Santa Rosa, CA, USA). The samples consisted in aerogel films with composition corresponding to samples FG, FG_NDs 0.5% and FG_NDs 1%, air-dried in an oven, at 40 °C. They were glued on the sample stage. The continuous stiffness measurement (CSM) was performed using a Berkovich tip, with 25 indentations with 50 µm distance between them (to prevent interactions between indentations) for each sample. Indentations were set to a maximum penetration depth of 2000 nm with a strain rate target of 0.05/s. This was achieved by superimposing a 2 nm of amplitude and 45 Hz of frequency small oscillating force during the loading cycle. In brief, an indentation test started with the indenter tip approaching the surface with an approach velocity of 10 nm/sec. When the indenter touched the surface, it started to penetrate the surface at a rate of 0.05 s^−1^. At the maximum penetration depth of 2000 nm, the load on the indenter was held constant for 10 s, then it was withdrawn from the sample. When the load on the sample reaches 10% of the maximum load on the sample, it was held constant for 100 s. The indenter was totally withdrawn, and the sample is moved for the next test.

### 2.3. Biocompatibility Evaluation of the Fibrous Scaffolds

To monitor the influence of the nanoparticles content, the FG_NDs nanocomposite meshes were tested for their biocompatibility in contact with human adipose-derived stem cells (hASCs). Cells were isolated from subcutaneous adipose tissue resulted after liposuction only after obtaining the informed consent of the patient. All performed studies that included hASCs were in compliance with the Helsinki Declaration, with the approval of the University of Bucharest Ethics Committee. In order to isolate stem cells, previously described protocol was applied [33,34]. Afterwards, the primary cell culture was maintained in Dulbecco’s Modified Eagle Medium (DMEM, Sigma-Aldrich Co, Steinheim, Germany) supplemented with 1% antibiotic and 10% fetal bovine serum (FBS) and grown until passage 4. The scaffolds were sterilized by exposure to UV light, cells were seeded at 2 × 10^4^ cells/cm^2^ and incubated for one week in standard conditions (37 °C, 5% CO_2_ and humidity). Biocompatibility assays were performed at 2, 4, and 7 days post-seeding and after 48 h actin F filaments were stained in order to evaluate cell adhesion. During quantitative biocompatibility studies, FG_NDs_0.5% and 1% composites were compared to pure FG considered as control and to the reference tissue culture plate (TCP) control.

To assess if hASCs maintain their metabolic activity when put in contact with FG_NDs, methylthiazolyldiphenyl tetrazolium bromide (MTT, Sigma-Aldrich Co, Steinheim, Germany) assay was performed. First, culture media was discharged and then MTT solution was put over the composites and incubated for 4h at dark in standard conditions. MTT solution was prepared at the recommended concentration of 1 mg/mL in DMEM lacking FBS. Then, the formed violet formazan crystals were solved with isopropanol and the obtained solution was measured at 550 nm using FlexStation3 Spectrophotometer (Molecular Devices, San Jose, CA, USA).

LDH assay was performed in order to assess if the materials exhibit significant toxicity on hASCs. Therefore, “In vitro toxicology assay kit lactate dehydrogenase based” TOX7 kit (Sigma Aldrich Co, Steinheim, Germany) was used and the test was performed following the manufacturer’s instructions and the final solution was measured at 490 nm using FlexStation3 Spectrophotometer (Molecular Devices, USA).

Live/Dead staining was accomplished by using Live/Dead kit (ThermoFisher Scientific, Foster City, CA, USA). The solution was prepared following the manufacturer’s instructions, after discharging the culture media, the solution was put in contact with the bioconstructs and incubated for 20 min in dark. Laser-scanning confocal microscope (Carl Zeiss LSM 710 system, Zeiss, Germany) was used for visualization and the obtained images were analyzed using corresponding Zeiss Zen 2010 software.

For cell adhesion investigation, F-actin filaments of hASCs were evidenced 48 h post-seeding. For fixation of the cells, a 4% paraformaldehyde solution (Sigma Aldrich Co, Steinheim, Germany) was used for 1 h. Then cell membrane was permeabilized with 0,1% Triton X-100 (Sigma Aldrich Co, Steinheim, Germany) solution in bovine serum albumin (BSA) for 45 min. Last, cell-scaffold systems were incubated for 1 h with phalloidin-FITC (Sigma Aldrich Co, Steinheim, Germany) and for 5 min with Hoechst 33342, in order to stain cell nuclei (ThermoFisher Scientific, Foster City, CA, USA). Laser-scanning confocal microscope (Carl Zeiss LSM 710 system, Zeiss, Germany) was used for visualization and the obtained images were analyzed using corresponding Zeiss Zen 2010 software. The quantification of the area covered by phalloidin-FITC (%) was made using Image J software (NIH, Bethesda, MD, USA, public software) as an average of several images for each composite (n = 10) and plots were obtained using GraphPad Prism version 3.

All experiments were performed in triplicate (n = 3) and the generated results were expressed as means ± standard deviation using GraphPad Prism 3.0 Software (GraphPad Software Inc., San Diego, CA, USA). The statistical relevance was assessed using this software, by performing one-way ANOVA and Bonferroni post-test, considering a statistical difference for *p* < 0.05.

## 3. Results

### 3.1. Rheological Evaluation of the Precursors

**Figure 1:**
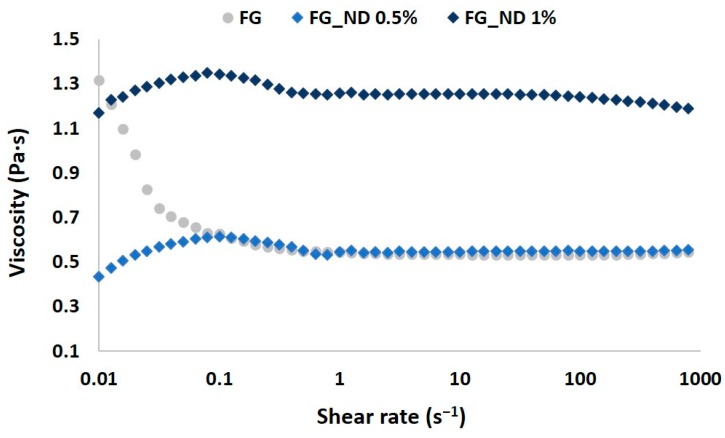
Graphical representation of the influence of precursors’ composition on the viscosity at different shear rates and room temperature.

To assess the composition effect on the viscosity of the precursors, steady state flow tests were performed at different shear rates at room temperature; the resulted dynamic viscosities of the solutions are shown in Figure 1.

Gelatin solution exhibited a shear thinning behavior at low shear rates when compared with the NDs loaded compositions which presented a slight shear thickening behavior for the same values of the shear rate. Furthermore, increasing shear rate above 1 s^−1^ a linear viscosity was recorded for FG and FG_ND 0.5%, the measured viscosity becoming independent of the shear rate. The FG_ND 1% composition presented higher viscosity compared to the samples FG and FG_ND 0.5%. One could explain this behavior as a consequence of both the increased concentration of the nanospecies within the solution, but also as a consequence of the higher amount of hydrogen bonds formed between the functional groups at the surface of the nanoparticles with the functional groups from the backbone of gelatin (NH_2_, COOH, OH) [35]. Moreover, another interesting aspect resulted from the measurements for FG_ND 1% is represented by the small tendency of decrease of the viscosity with increasing the shear rate (values over 80 s^−1^). This pseudoplastic behaviour may appear due to a more efficient dispersion of NDs through the polymer chains. However, the higher NDs content lead to a 2.3 times increase of the viscosity (from 0.54 Pa·s to 1.24 Pa·s). This behavior suggests the reinforcing potential of the nanoparticles on the fibrous scaffolds at 1% loading ratio. Such rheologycal comportment of the precursors were in agreement with previous results of our group [36].

### 3.2. Wettability

**Figure 2:**
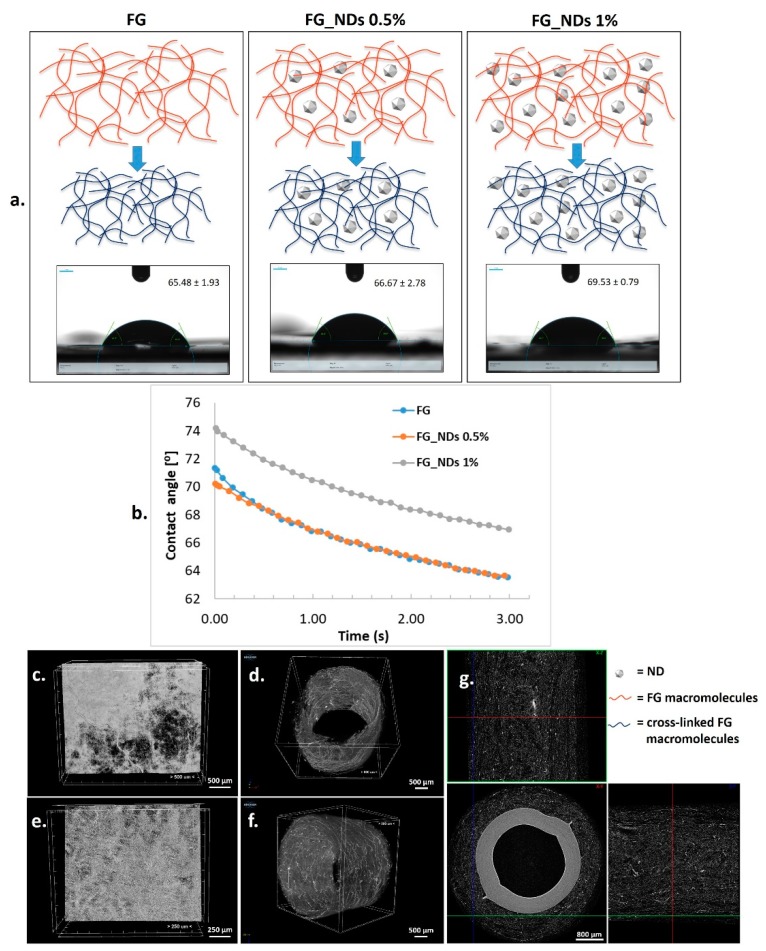
(**a**) Schematic representation of nanodiamond particles (NDs) nanocomposite synthesis from precursors, through fish gelatin (FG) cross-linking (red—FG macromolecules; blue—cross-linked FG macromolecules) (shrinkage during cross-linking is suggested; influence of the NDs content on the wettability expressed as contact angle results; micro-CT images of: (**b**) wettability evolution monitored during 3 s; (**c**) FG_NDs 0.5% mesh; (**d**) FG_NDs 0.5% rolled mesh; (**e**) FG_NDs 1% mesh; (**f**) FG_NDs 1% rolled mesh; (**g**) Data Viewer images of the microfibrous FG_NDs 1% rolled mesh; inner plastic support is visible as a compact ring.

**Figure 3:**
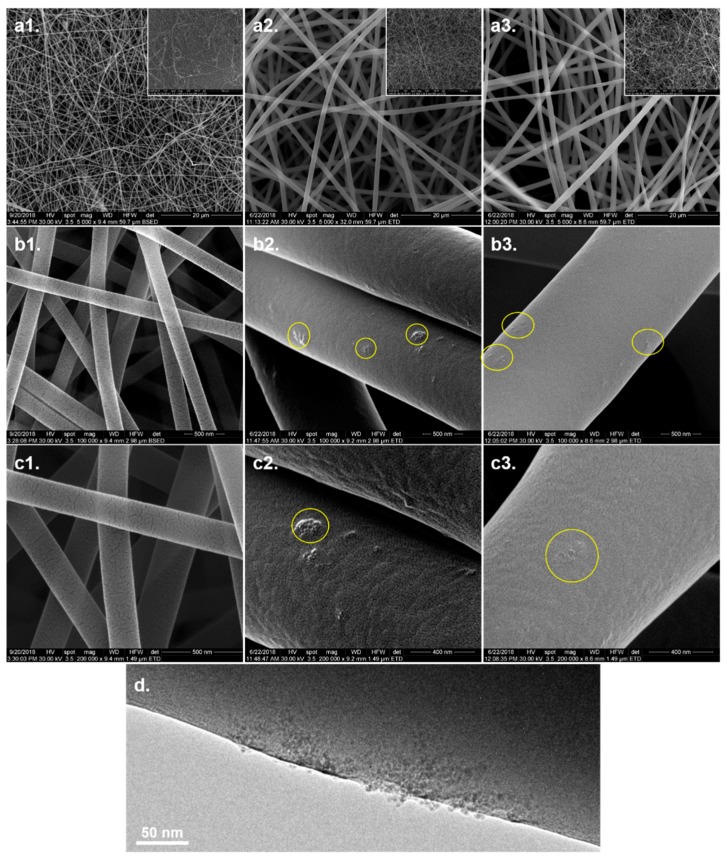
SEM images of electrospun fibers: (**a1**–**c1**) FG at different magnifications, (**a2**–**c2**) FG_ND 0.5% at different magnifications, (**a3**–**c3**) FG_ND 1% at different magnifications. (**d**) TEM micrograph of FG_ND 1%—functionalized NDs clusters in a fiber.

The nanocomposite meshes are obtained after FG cross-linking in the presence of NDs. The formation of cross-links occurs with shrinkage of the biopolymer due to the reduction of the distance following generation of new chemical bonds. Figure 2a. is representative with respect to the network shrinkage during the cross-linking process. The contact angle experiments revealed a slight modification in the average values, increasing from a value of approximately 66° for FG to 70° for the nanocomposite loaded with 1% NDs. This is also visible in Figure 3b. No significant difference has been noticed between the wettability of pristine FG and the nanocomposite containing 0.5% NDs (Figure 2a,b).

### 3.3. Microstructural Analysis

MicroCT imaging provided an overview of the microfibrous structure of the cross-linked electrospun scaffolds and of the tubular constructs that can be generated after rolling the meshes on polypropylene collectors. Representative images are given in Figure 2c–g, presenting the potential of this simple method to obtain tubular scaffolds which could be used for nerve guidance channels.

The microstructure of the scaffolds has been further investigated by SEM, HRSEM, and TEM. The electrospun meshes did not exhibited significant differences regarding the microstructure. All compositions led to fabrication of homogeneous fibers, continuous, with smooth surface, without defects along their length, entangled into mats with interconnected porosity. The average diameter of the fibers is of approximately 0.2 μm for FG, ~1.1 μm for FG_ND 0.5%, and ~1 μm for FG_ND 1% (with a larger diameter distribution) as one can observe in Figure 3(a1–a3). Figure 3(b2,b3) presented a rather uniform distribution of the nanoparticles into the polymeric fibers along their axes, in the form of nanoclusters. A higher tendency of cluster formation could be observed for the FG_ND 0.5% composition (as it can be seen in Figure 3(c2,c3)). TEM micrograph provided information on the distribution of the nanoparticles into the fibers. It can be observed that the nanoparticles are immobilized into the polymeric matrix, with a protruded aspect at the surface of the fiber. However, the NDs are not homogeneously dispersed into the fiber, rather presenting a tendency to form nanoparticles agglomerates along the axes of the fibers (Figure 3d). The crystalline phase distinguishes from the amorphous glassy continuous matrix of the FG fibers; it is obviously different from the point of view of the mechanical properties. Such a distribution of the nanoparticles was expected due to the low content of nanoparticles used in this work and it is in agreement with previous results of our group [19].

### 3.4. Nanomechanical Investigation

**Figure 4:**
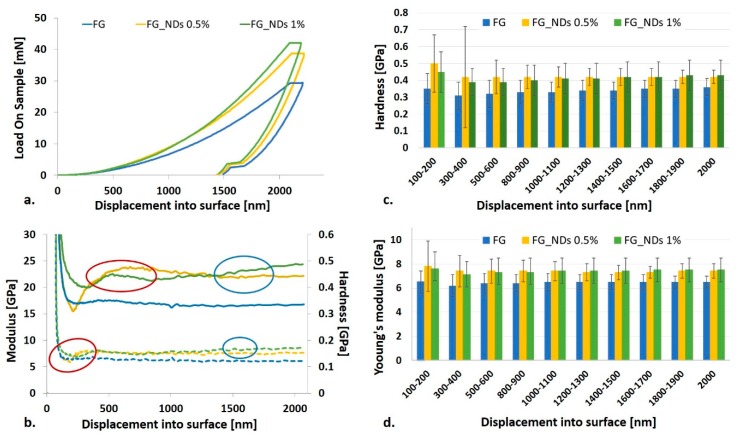
Influence of NDs loading on the nanomechanical properties of FG_NDs (**a**) load as function of penetration depth; (**b**) representative variation of modulus (top—dotted lines) and hardness (bottom—plain lines) up to a maximum depth of 2000 nm; red circles—the effect on FG-ND 0.5% which could be attributed to the presence of nanoparticles aggregates; blue circles—the expected behavior which appear with indentation depth increase; (**c**) hardness as a function of the indentation depth; (**d**) Young’s modulus as a function of the indentation depth.

Nanomechanical sensing influences the behavior of cells, therefore it became appealing to investigate how the mechanical properties at the nanoscale are influenced by the NDs content, at such low loadings. Nanoindentation can discriminate between similar, low-modulus samples when the composition is modified, and therefore we selected it as a method to explore the potential reinforcement effect of NDs nanoparticles, at low concentrations such as 0.5% and 1%. Preliminary characterization of dried FG_NDs scaffolds has been performed using the CSM based method as previously reported [37,38,39,40], and results were calculated according to Oliver and Pharr’s method [41]. This method allows a continuous measuring of the stiffness throughout the indentation’s loading cycle, allowing the calculation of E and H as a function of the indentation depth. Load is monitored as function of the displacement (Figure 4a), contributing to a better understanding of the nanomechanical behavior. This analysis revealed that the maximum load required to reach the same penetration depth increases with increasing NDs content. It has been noticed that during the 10 s peak hold time, a creep deformation appears regardless the sample’s composition. The plastic deformation is also shown by the curves in Figure 4a.

The variation of modulus and hardness with the penetration depth are displayed in Figure 4b–d. One can observe that results are more homogeneous with increasing the penetration depth, most probably due to the decrease of the nanomechanical heterogeneity in the dried samples from surface to bottom. The solvent evaporation during the samples’ preparation leads to a shrinkage of the polymer material with a significant change in the resulting elasticity. If in the hydrated compositions, the reinforcing effect of the NDs has been emphasized by a significant increase in the viscosity as measured by rheology, it was expected that the dried samples to present only minor differences in terms of stiffness since they correspond to a total solid content of 50.5% for FG_NDs 0.5% and respectively 51% for FG_NDs 1%, respectively. The big standard deviations (Figure 4c,d) for the displacement range from 100 nm to 200 nm are due to the surface anomalies which, once with the increase of indentation depth have a lower influence. Table 1 summarizes the increase of the values for Young’s modulus and hardness calculated as percentage related to the values obtained for simple FG. These values clearly suggest the decrease of both Young’s modulus and hardness with increasing the indentation depth.

The values of the Young’s modulus recorded for pure FG are ranging between 6.2 and 6.5 GPa on the entire indentation depth interval, when compared with FG_ND 0.5% for which the Young’s modulus values are ranging between 7.3–7.8 GPa, and for FG_ND 1% the values of the Young’s modulus are ranging between 7.1–7.6 GPa.

This observation is supported by the SEM and TEM micrographs (Figure 3) identifying agglomerated nanoparticles dispersed into the amorphous polymer fibers. While NDs are crystalline materials, the dried FG matrix is in a glassy state and accordingly, the resulting local mechanical heterogeneity can be discriminated by nanoindentation. Increasing the NDs content from 0.5% to 1% enhances the homogeneity of the nanoparticles’ dispersion in the FG_NDs 1% nanocomposite. In the case of FG_NDs 0.5%, the higher values obtained for both modulus and hardness with significantly high standard deviations than for FG_ND1% at lower indentation depth, could be explained by the presence of nanoparticles aggregates closer to the surface.

### 3.5. FT-IR Analysis

**Figure 5:**
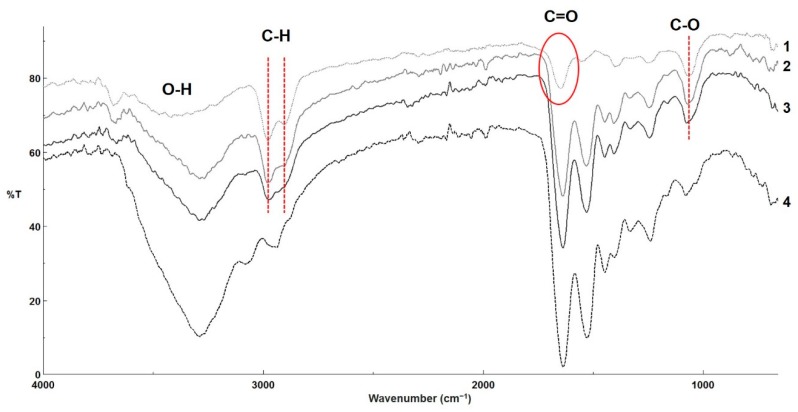
FT-IR spectra recorded on control samples: 1—NDs, and 4—FG and on the fibrous scaffolds 2—FG_ND 1%, 3—FG_ND 0.5% (increasing amount of NDs intensifies the specific vibrations characteristic for this component).

FT-IR analysis confirmed the presence of characteristic functional groups from the two individual components of the fibrous scaffolds. Functionalized NDs (Figure 5(1)) present a large band between 1700 and 1800 cm^−1^, attributed to C=O stretching in carboxylic groups. A peak related to C-O stretching was also visible at 1066 cm^−1^, attributed to the presence hydroxyl group (confirmed by the large band between 3000 and 3600 cm^−1^ due to O-H stretching) and/or ether groups on the surface of the nanoparticles [19,42]. C–H stretching was also present between 2800 and 3000 cm^−1^, linked to a very limited amount of amorphous carbon lying on the surface of the NDs. FG (Figure 5(4)) presents specific vibrations such as broad peak at 3289 cm^−1^, which is common signal for O–H and N–H stretching, a small peak at 3082 cm^−1^ attributed to N–H, at 2938 cm^−1^ is present the specific vibration for saturated C–H stretch, and amide I peak at 1637 cm^−1^ and amide II peak at 1531 cm^−1^. For the nanocomposite fibrous scaffolds, all the specific vibrations of the component can be noticed in the correspondent spectrum (Figure 5(2) depicts the IR spectrum for FG_ND 1%, and Figure 5(3) depicts the FT-IR spectrum for FG_ND 0.5%).

### 3.6. Biocompatibility Assessment

**Figure 6:**
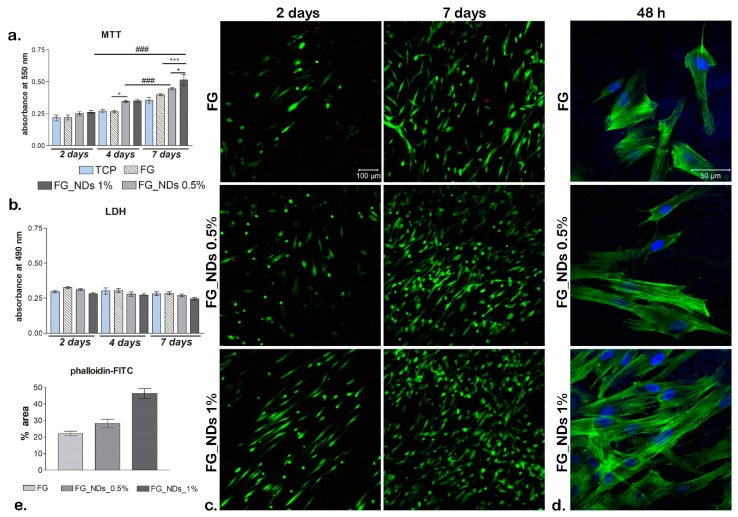
Biocompatibility and adhesion assays performed for hASCs/FG_NDs bioconstructs (**a**) Cell viability profile obtained after one week of culture by MTT test. Statistical significance: * *p* < 0.05; *** and ### *p* < 0.001. (**b**) Cytotoxicity levels exerted by FG_NDs on hASCs during 7 days of culture (**c**) Cell viability and proliferation qualitative analysis obtained after performing Live/Dead staining on the tested composites; live—green labeled with calcein AM and cell nuclei of dead cells—red labeled with ethidium bromide (**d**) F-actin filaments developed by hASCs in contact with FG_NDs after 48h of culture; filaments (green) are stained in phalloidin-FITC, while nuclei (blue) are stained with Hoechst 33342. (**e**) quantification of phalloidin-FITC levels in all controls.

MTT profile (Figure 6a) indicateds, at 2 days post-seeding, that cell viability was the same on the two nanocomposites containing NDs as compared to the FG control and TCP control. On the other hand, at 4 days a significant (*p* < 0.05) difference was found between FG_NDs nanocomposites and FG, and no difference in terms of viability between FG_NDs 0.5% and FG_NDs 1%. After 7 days of culture, most significant (*p* < 0.001) proportion of viable cells were found in contact with FG_NDs 1% as compared to the FG control. Moreover, a relevant (*p* < 0.05) difference was also found on FG_NDs 0.5% as compared to FG_NDs 1%. No significant proliferation occurred from 2 to 4 days post-seeding on the tested composites. In contrast, proliferation rate was found to be higher for 4 to 7 days of culture, with a significant (*p* < 0.001) increase on the material loaded with 0.5% NDs. Also, a big increase in proliferation can be observed from 2 to 7 days on all tested composites, but with a significant (*p* < 0.001) relevance for the nanocomposite scaffold containing 1% NDs. This behavior can be correlated with the previously described nanomechanical properties. Therefore, it can be concluded that the addition of NDs in the fibrous hydrogel scaffolds supports cell viability and increases the proliferation rate.

LDH assay (Figure 6b) revealed overall small amounts of toxicity during 7 days of culture. Moreover, it can be observed how these remained constant from 2 days until 7 days post-seeding. Also, levels of LDH tend to be lower (but not significantly) on composites enriched with NDs as compared to the tested FG control and similar to the levels registered for TCP control. Thus, the presence of NDs in these materials did not induce significant cytotoxicity on hASCs.

By simultaneously labeling live (green) and dead (red) cells (Figure 6c), it can be noticed that there is a significant proportion between live and dead cells on all tested composites. Images obtained at the confocal microscopy revealed that cells proliferated and maintained their viability throughout one week of culture, thus confirming MTT and LDH results. Within 2 days of culture, more viable cells are to be observed on FG_NDs 1% as compared to the control and FG_NDs 0.5%. Moreover, hASCs in contact with FG_NDs 1% present a different morphology, they are elongated as compared to FG_NDs 0.5% where most of them are still in their round shape. After 7 days, a significant amount of cells is to be evidenced on all nanocomposites, but when it comes to composites enriched with NDs, hASCs have formed groups, this meaning that these particular scaffolds offered them optimal conditions for proliferation.

After cell cytoskeleton was evidenced by staining with phalloidin-FITC (Figure 6d), a good cell adhesion was observed on the investigated nanocomposites. There are no significant changes in cell phenotype from a material to another. Long actin fibers were found on all composition, confirming that hASCs were able to develop cytoskeleton in contact with FG_NDs, similar to the one formed in contact with FG. However, F-actin filaments are clearly more visible on FG_NDs 1% than in case of FG_0.5% and the control, this meaning that after 48 h hASCs had a better interaction with this particular scaffold. Hereby this is another confirmation that incorporation of NDs in the structure of biomaterials has a positive impact on behavior of mesenchymal stem cells.

## 4. Discussion

In the last years, the scientists tried to better understand the interaction processes between a biomaterial and the living cells through nanomechanics responses induced by nano-cues loaded in commonly used biopolymers for TE applications. A deeper understanding of the small scale phenomena that have a great influence on the cells behavior (either positive [19,43] in stimulating cellular adhesion, proliferation, differentiation, or negative [44] through the alteration of the cells’ properties which eventually leads to important conditions), could provide suitable solutions in case of TE and cellular therapy [16]. Due to the continuous need for improvement, natural polymers, nanotechnologies, and stem cells seem to hold a great promise for generating substitutes that could guide an injured tissue towards regeneration.

Based on our group previous studies [19,36] we synthesized randomly oriented multifunctional electrospun composites scaffolds based on FG and NDs and we thoroughly characterized them regarding physico-chemical and biocompatibility properties. In order to avoid the common tendency of the NDs to create aggregates, we have used a mild sonication treatment of the NDs dispersions in aqueous FG solutions during the obtaining of the fibers’ precursors. Consistent with previous researches [35,36], the rheological characterization of the precursors provided information regarding the viscosity of the mixture. As expected, no significant differences were observed between the simple FG and FG_ND 0.5% solutions, but for FG_ND 1% precursor 2.3 times increase of the viscosity was recorded. The behavior could be explained due to interactions between the functional groups from the surface of NDs and the functional groups of the gelation macromolecules, in accordance with other studies [35,36,45]. The effect of the NDs can be slightly observed on the increased hydrophobicity of FG_ND 1% material. In their study, Mahdavi et al., 2016 [22], fabricated through electrospinning composite scaffolds based on chitosan, bacterial cellulose, and NDs. They observed a similar behavior regarding the wettability of their fibers with the behavior of our mats. They suggested that even though the NDs present many hydrophilic functional groups on their surface, a bigger influence in the final wettability properties it is given by the surface energy and the surface geometrical features of the material. One could explain the behavior of our materials through the same mechanisms, meaning that an increase of the NDs loading is leading to a decrease of the wettability [46,47].

An important repercussion of the viscosity of the precursors was further reflected in the influenced over the morphology of the fibers as shown in SEM micrographs. The introduction of the NDs increased considerably the diameter of the fibers. It is possible that the effect of the NDs is related to the enhanced viscosity and thus, changes of the conductivity could appear due to the formation of weak physical cross-linking bonds between the NDs and the FG. In a previous study of Li et al., 2016 [48], it was shown that increased viscosity of the solution translates in a weaker stretching of the liquid jet during the electrospinning process, which can lead to an increase of the diameter of the fabricated fibers. Also, Pacelli et al. 2017, have shown that the injectability of chitosan hydrogels loaded with NDs is affected because higher NDs concentration leads to a high number of polar functional groups in the gel [49]. This behavior, associated with the tendency of the nanoparticles to form clusters, could be an explanation of the bigger diameters of the nanocomposite fibers. Also, since the NDs can act as cross-linking points, the macromolecular coil of gelatin might not stretch enough to permit the fabrication of thin fibers, as in the case of the simple FG solution. These results presented in the literature can be correlated with the rheological characterization of our precursors and the diameter of the resulting fibers, namely, the superficial cross-liking between NDs and gelatin led to thicker but less homogeneous dimensions of the fibers. Regarding the distribution of the NDs within the fibers, our findings agree with previous studies of our group [19,36]. Thus, the NDs do not present a homogeneous distribution along the fibers, but they are rather forming clusters, present predominately at the surface of the fibers. We anticipated that the superficial nanostructuring of the surface of the fibers will have an important influence on the cellular behavior, as will further be discussed.

In our experiment, the bioconstruct consisting of natural polymer material—FG, loaded with NDs, and cultured with hASCs exhibited an overall good biocompatibility and promising potential as a support platform for TE and regenerative medicine. The main finding of this study is the ability of hASCs to respond differently to the variable composition of the FG_NDs composites, particularly depending on the NDs concentration within the materials. In other words, the inclusion of NDs in these fibrous gelatin electrospun meshes appears to be a useful tool for the modulation of the cellular component behavior in terms of cell growth, proliferation and adhesion.

As previously stated, gelatin has a great potential in serving as a substrate for cell growth within TE applications. Its potential for various directions in the field of TE has been proven in a recent study, where cells were encapsulated within the material. It seems that alginate/gelatin microspheres which contained hASCs have supported cell viability, proliferation and adipogenic differentiation [50], therefore demonstrating gelatin’s favorable effect on cell behavior. As a consequence, in our study gelatin represented the base of the scaffold. Particularly, fish gelatin was used because it has low gelation temperature, which permitted the production of the fibers at room temperature from very concentrated aqueous solution, comparing with mammalian gelatins, thus, providing more similarities with the ECM [51]. As MTT profile revealed, there is no significant difference in cell proliferation from day 2 to day 7 post-seeding, but at the same time, no significant toxicity levels were found, therefore proving that gelatin is biocompatible with hASCs.

In order to enhance a materials’ ability to promote cell proliferation, recent biomaterial development includes addition of nanoparticles in order to obtain optimal cell-scaffold interaction [17]. NDs are known for their exquisite properties and good interaction with cells and this has been proven several times in different studies. Previous findings indicated that treating lung epithelial cells and normal fibroblasts with a suspension of carboxylated NDs did not affect cell viability and cells did not undergo apoptosis, whereas nanotubes induced cytotoxicity in the same cell types, thus demonstrating that NDs are less toxic than other carbon-based structures used in the field of TE [52]. Moreover, it has been shown that these nanoparticles would not affect stem cells, the key players in regeneration. It has been demonstrated that including NDs to poly (L-lactide-co-ε-caprolactone) (PCL) scaffolds promoted cell proliferation and differentiation of human bone marrow-derived mesenchymal stem cells (hBM-MSCs) towards osteogenic lineage [21]. Moreover, data indicated that presence of NDs did not affect viability of human mesenchymal stem cells and stimulated their migration on the materials surface [53]. Therefore, NDs have a non-toxic effect on stem cells and highly promote their growth and proliferation on scaffolds. Similar to these observations, our biocompatibility results demonstrated that incorporation of NDs in the material structure had a benefic impact on hASCs cell culture in terms of viability, proliferation and adhesion. MTT profile revealed that cells maintained their viability and started to proliferate during 7 days of culture in contact with these composites. It was observed how the addition of NDs significantly increased the proliferation rate of hASCs from 4 to 7 days post-seeding (*p* < 0.001). LDH assay revealed no significant levels of cytotoxicity, thus demonstrating that NDs presence in the scaffold did not exhibit any negative impact upon hASCs. Interestingly, the staining of live cells by Live/Dead assay revealed that these stem cells already presented an equal distribution at 2 days post-seeding on FG_ND 1% surfaces, whereas, for the control FG and FG_ND 0.5%, smaller cell groups could be observed. The abundant groups of cells formed after 7 days of culture on both materials containing NDs demonstrating that the material continued to support cell viability and proliferation on the long term. This proves that incorporation of these nanoparticles enhances a good interaction between stem cells and nanocomposite fibrous scaffolds. These findings together with the microstructural and nanomechanical data suggest that it is likely that the nanocomposite clusters dispersed at smaller distance within the FG_NDs 1% fibers with respect to the 0.5% samples contribute through mechanical sensing to the guidance of cells.

The results of the nanoindentation tests come to support and explain the cell behavior. Thus, the results obtained for displacement range from 100 nm to 200 nm presented big standard deviations, phenomenon that can be explained by the heterogenous aspect of the surface. Higher indentation depth, not only that led to lower standard deviation but also to lower values for both Young modulus and hardness. The observation, supported both by SEM and TEM analysis, indicate the tendency of NDs to locate and form agglomerates at the surface of the fibers. These clusters formed at the surface of the fibers can act as guidance cues for cells in order to better adhere, migrate and further proliferate at the interface with the biomaterial.

Cell adhesion is a critical step in the evaluation of cell-scaffold interaction since cell surface receptors interact directly with the components and surface of the substrate. Therefore, it is important for the material to own specific proprieties that allow cells to easily attach to its surface. Recent work has established that gelatin generates a quite similar structure to the natural ECM, therefore cells adhere with ease to these surfaces. Fibrous structures usually tend to provide bigger areas and to promote cell adhesion and growth than other materials with a smooth surface. Guarino et al. [54] showed that human mesenchymal stem cells had a superior interaction with PCL membranes when gelatin was added to their structure. Besides, NDs have also shown to enhance cell adhesion due to their favorable physical proprieties [17,18]. Pereia et al. [55] fabricated a poly (lactic acid) scaffold which contained NDs and investigated biological and bioactive proprieties of this electrospun fiber. The conclusion of their study was that incorporation of these particles to the materials structure delivered satisfying cell-scaffold interaction. In our study, marking F-actin filaments after 48 h of culture, exposed an interesting behavior of hASCs in contact with these meshes, namely the elongation of the cytoskeleton increasing with NDs concentration in the material. We have concluded that the presence of NDs delivers a good impact on cell behavior, in terms of stimulating hASCs attachment to the scaffold and allowing cells to grow and multiply. This study confirmed that most favorable hASCs response was registered in contact with FG_NDs 1%, suggesting once more that a better cell-scaffold interaction occurs dependent on NDs’ ratio in the final composite.

Even though lots of studies involving NDs are focusing on bone and cartilage tissue engineering, recent work has demonstrated NDs potential to support neuron attachment and neurite outgrowth, therefore making these particles potential candidates for nervous tissue engineering. Hopper et al. [56] studied the effect of NDs on NG108-15 cell line and on primary dorsal root ganglion (DRG) neurons, concluding that NDs promoted neuronal cell adhesion, migration and neurite outgrowth. Moreover, due to gelatin’s hydrophilic surface and high water content, it has been demonstrated that this kind of material supported hASCs differentiation towards neural*-like* cell types, expressing specific markers as nestin and Tuj-1 [57]. All these findings together with our results confirm that functionalizing gelatin meshes with NDs and together with hASCs hold a great promise for more sophisticated applications in TE, such as peripheric nerve regeneration. Therefore, we envisage developing further studies where to analyze hASCs potential to differentiate towards neural or glial cell types, assisted by FG_NDs platforms that show ability to support this TE direction. Preliminary data shows that these substrates are capable to promote survival and growth of primary DRG neurons, therefore expanding the advantages of NDs to this new field of nerve regeneration.

## 5. Conclusions

An overall enhanced interaction between hASCs and FG_NDs revealed that cell viability and proliferation were not diminished by the incorporation of NDs up to 1% and confirmed low cytotoxicity of the obtained nanocomposite meshes. The main hypothesis of this study consisted in the potential modulation of cell response by NDs incorporation into the fibrous gelatin meshes. One of the most important monitored effects was the modification of the nano, micro-structure of the fibers surface and, accordingly, of the stiffness due to the increasing yet low nanoparticle content. Nanoindentation data combined with the microstructural analyses suggested a local increase of the scaffolds’ stiffness more homogenous in the samples loaded with 1% NDs, due to a better distribution of small clusters along the fibers’ longitudinal axis. Such behavior can be considered a proof for a nanomechanical sensing that can explain the modification of cell morphology, adhesion, and proliferation with the best results for the highest nanoparticle loading. Overall, hASCs displayed different behavior in response to increasing NDs loading in the FG_NDs composites, thus confirming that their use for different regenerative applications can be modulated by such tools. These findings and NDs potential have to be explored in the near-future to better understand the interaction between hASCs and the nano-components, in order to extend hASCs applications in other fields than the already confirmed ones - bone, cartilage, and adipose TE. Considering this idea and NDs potential for nerve regeneration, FG_NDs capability to support hASCs differentiation to neuronal cell types and consequent peripheric nerve reconstruction post-injury should be further investigated.

## Figures and Tables

**Table 1:** The percent of mechanical properties increase with addition of NDs in respect to the values obtained for FG.

Indentation Depth [nm]	Young’s Modulus (%)		Hardness (%)	
	FG_NDs 0.5%	FG_NDs 1%	FG_NDs 0.5%	FG_NDs 1%
100–200	20.00	16.92	42.86	28.57
300–400	19.35	14.52	35.48	25.81
500–600	15.63	14.06	31.25	21.88
800–900	15.63	14.06	27.27	21.21
1000–1100	13.85	13.85	27.27	24.24
1200–1300	12.31	13.85	23.53	20.59
1400–1500	12.31	13.85	23.53	23.53
1600–1700	12.31	15.38	20.00	20.00
1800–1900	13.85	15.38	20.00	22.86
2000	13.85	15.38	16.67	19.44

## References

[1] Dzobo K., Thomford N.E., Senthebane D.A., Shipanga H., Rowe A., Dandara C., Pillay M., Motaung K.S.C.M. (2018). Advances in regenerative medicine and tissue engineering: Innovation and transformation of medicine. Stem Cells Int..

[2] Salgado A.J., Oliveira J.M., Martins A., Teixeira F.G., Silva N.A., Neves N.M., Sousa N., Reis R.L., Stefano G., Isabelle P., Pierluigi T., Bruno B. (2013). Tissue engineering and regenerative medicine: Past, present, and future. Title of International Review of Neurobiology.

[3] Sell S.A., Wolfe P.S., Garg K., McCool J.M., Rodriguez I.A., Bowlin G.L. (2010). The use of natural polymers in tissue engineering: A focus on electrospun extracellular matrix analogues. Polymers.

[4] Karim A.A., Bhat R. (2009). Fish gelatin: Properties, challenges, and prospects as an alternative to mammalian gelatins. Food Hydrocolloid.

[5] Shakila R.J., Jeevithan E., Varatharajakumar A., Jeyasekaran G., Sukumar D. (2012). Comparison of the properties of multi-composite fish gelatin films with that of mammalian gelatin films. Food Chem..

[6] Yoon H.J., Shin S.R., Cha J.M., Lee S.H., Kim J.H., Do J.T., Song H., Bae H. (2016). Cold water fish gelatin methacryloyl hydrogel for tissue engineering application. PloS ONE.

[7] Lee K.Y., Mooney D.J. (2001). Hydrogels for tissue engineering. Chem. Rev..

[8] Afewerki S., Sheikhi A., Kannan S., Ahadian S., Khademhosseini A. (2019). Gelatin-polysaccharide composite scaffolds for 3D cell culture and tissue engineering: Towards natural therapeutics. Bioeng. Transl. Med..

[9] Manikandan A., Thirupathi Kumara Raja S., Thiruselvi T., Gnanamani A. (2018). Engineered fish scale gelatin: An alternative and suitable biomaterial for tissue engineering. J. Bioact. Compat. Polym..

[10] Fu C., Bai H., Hu Q., Gao T., Bai Y. (2017). Enhanced proliferation and osteogenic differentiation of MC3T3-E1 pre-osteoblasts on graphene oxide-impregnated PLGA–gelatin nanocomposite fibrous membranes. Rsc Adv..

[11] Lai J.Y., Li Y.T., Cho C.H., Yu T.C. (2012). Nanoscale modification of porous gelatin scaffolds with chondroitin sulfate for corneal stromal tissue engineering. Int. J. Naonomed..

[12] Huber B., Borchers K., Tovar G.E., Kluger P.J. (2016). Methacrylated gelatin and mature adipocytes are promising components for adipose tissue engineering. J. Biomater. Appl..

[13] Ghasemi-Mobarakeh L., Prabhakaran M.P., Morshed M., Nasr-Esfahani M.H., Ramakrishna S. (2008). Electrospun poly (ε-caprolactone)/gelatin nanofibrous scaffolds for nerve tissue engineering. Biomaterials.

[14] Yamamoto M., Tabata Y., Ikada Y. (1999). Growth factor release from gelatin hydrogel for tissue engineering. J. Bioact. Compat. Polym..

[15] Houshyar S., Kumar S., Rifai A., Tran N., Nayak R., Shanks R.A., Padhye R., Fox K., Bhattacharyya A. (2019). Nanodiamond/poly-ϵ-caprolactone nanofibrous scaffold for wound management. Mat. Sci. Eng. C-Mater..

[16] Chen J. (2014). Nanobiomechanics of living cells: A review. Interface Focus.

[17] Bacakova L., Broz A., Liskova J., Stankova L., Potocky S., Kromka A., Mahmood A. (2016). The application of nanodiamond in biotechnology and tissue engineering. Title of Diamond and Carbon Composites and Nanocomposites.

[18] Bacakova L., Grausova L., Vandrovcova M., Vacik J., Frazcek A., Blazewicz S., Kromka A., Rezek B., Vanecek M., Nesladek M., Simone L.L. (2008). Carbon nanoparticles as substrates for cell adhesion and growth. Title of Nanopartiles: New Research.

[19] Serafim A., Cecoltan S., Lungu A., Vasile E., Iovu H., Stancu I.C. (2015). Electrospun fish gelatin fibrous scaffolds with improved biointeractions due to carboxylated nanodiamond loading. Rsc Adv..

[20] Eivazzadeh-Keihan R., Maleki A., de la Guardia M., Bani M.S., Chenab K.K., Pashazadeh-Panahi P., Baradaran B., Mokhtarzadeh A., Hamblin M.R. (2019). Carbon based nanomaterials for tissue engineering of bone: Building new bone on small black scaffolds: A review. J. Adv. Res..

[21] Xing Z., Pedersen T.O., Wu X., Xue Y., Sun Y., Finne-Wistrand A., Kloss F.R., Waag R., Krueger A., Steinmuller-Nethl D. (2013). Biological effects of functionalizing copolymer scaffolds with nanodiamond particles. Tissue Eng. Part. A.

[22] Mahdavi M., Mahmoudi N., Anaran F.R., Simchi A. (2016). Electrospinning of Nanodiamond-Modified Polysaccharide Nanofibers with Physico-Mechanical Properties Close to Natural Skins. Mar. Drugs.

[23] Thalhammer A., Edgington R.J., Cingolani L.A., Schoepfer R., Jackman R.B. (2010). The use of nanodiamond monolayer coatings to promote the formation of functional neuronal networks. Biomaterials.

[24] Yu G., Floyd Z.E., Wu X., Hebert T., Halvorsen Y.D.C., Buehrer B.M., Gimble J.M., Gimble J., Bunnell B. (2011). Adipogenic Differentiation of Adipose-Derived Stem Cells. Title of Adipose-Derived Stem Cells. Methods in Molecular Biology (Methods and Protocols).

[25] Grottkau B.E., Lin Y. (2013). Osteogenesis of adipose-derived stem cells. Bone Res..

[26] Oh S.J., Park H.Y., Choi K.U., Choi S.W., Kim S.D., Kong S.K., Cho K.S. (2018). Auricular Cartilage Regeneration with Adipose-Derived Stem Cells in Rabbits. Mediat. Inflamm..

[27] Ferroni L., Gardin C., Tussardi I.T. (2012). Potential for Neural Differentiation of Mesenchymal Stem Cells. Adv. Biochem. Eng. Biotechnol..

[28] Jahan-Abad A.J., Morteza-Zadeh P., Negah S.S., Gorji A. (2017). Curcumin attenuates harmful effects of arsenic on neural stem/progenitor cells. Avicenna J. Phytomed..

[29] Krishna L., Dhamodaran K., Jayadev C., Chatterjee K., Shetty R., Khora S.S., Das D. (2016). Nanostructured scaffold as a determinant of stem cell fate. Stem Cell Res..

[30] Wang Z., Ruan J., Cui D. (2009). Advances and prospect of nanotechnology in stem cells. Nanoscale Res. Lett..

[31] Huang T., He D., Kleiner G., Kuluz J. (2007). Neuron-like Differentiation of Adipose-Derived Stem Cells From Infant Piglets in Vitro. J. Spinal Cord Med..

[32] Darling E.M., Topel M., Zauscher S., Vail T.P., Guilak F. (2008). Viscoelastic properties of human mesenchymally-derived stem cells and primary osteoblasts, chondrocytes, and adipocytes. J. Biomech..

[33] Galateanu B., Dinescu S., Cimpean A., Dinischiotu A., Costache M. (2012). Modulation of Adipogenic Conditions for Prospective Use of hADSCs in Adipose Tissue Engineering. Int. J. Mol. Sci..

[34] Dinescu S., Galateanu B., Albu M., Cimpean A., Dinischiotu A., Costache M. (2013). Sericin enhances the bioperformance of collagen-based matrices preseeded with hADSCs. Int. J. Mol. Sci..

[35] Zhang Y., Hua Q., Zhang J.M., Zhao Y., Yin H., Dai Z., Zheng L., Tang J. (2018). Enhanced thermal and mechanical properties by cost-effective carboxylated nanodiamonds in poly (vinyl alcohol). Nanocomposites.

[36] Cecoltan S., Serafim A., Dragusin D.-M., Lungu A., Lagazzo A., Barberis F., Stancu I.-C. (2016). The potential of NDPs-loaded fish gelatin fibers as reinforcing agent for fish gelatin hydrogels. Key Eng. Mater..

[37] Woo D.J., Sneed B., Peerally F., Heer F.C., Brewer L.N., Hooper J.P., Osswald S. (2013). Synthesis of nanodiamond-reinforced aluminum metal composite powders and coatings using high-energy ball milling and cold spray. Carbon.

[38] Hardiman M., Vaughan T.J., McCarthy C.T. (2015). Fibrous composite matrix characterisation using nanoindentation: The effect of fibre constraint and the evolution from bulk to in-situ matrix properties. Compos. Part. A Appl. Sci. Manuf..

[39] Behler K.D., Stravato A., Mochalin V., Korneva G., Yushin G. (2009). Nanodiamond-Polymer Composite Fibers and Coatings. Asc Nano.

[40] Lee S., Teramoto Y., Wang S., Pharr G.M., Rials T.G. (2006). Nanoindentation of Biodegradable Cellulose Diacetate- graft -Poly (L -lactide) Copolymers: Effect of Molecular Composition and Thermal Aging on Mechanical Properties. J. Polym. Sci. Polym. Phys..

[41] Oliver W.C., Pharr G.M. (1992). An improved technique for determining hardness and elastic modulus using load and displacement sensing indentation experiments. J. Mater. Res..

[42] Kim Y., Lee D., Kim S.Y., Kang E., Kim C.K. (2019). Nanocomposite Synthesis of Nanodiamond and Molybdenum Disulfide. Nanomaterials.

[43] Huang Y.A., Kao C.W., Liu K.K., Huang H.S., Chiang M.H., Soo C.R., Chang H.C., Chiu T.W., Chao J.I., Hwang E. (2014). The effect of fluorescent nanodiamonds on neuronal survival and morphogenesis. Sci. Rep..

[44] Lee G.Y.H., Lim C.T. (2007). Biomechanics approaches to studying human diseases. Trends Biotechnol..

[45] Sun Y., Yang Q., Wang H. (2016). Synthesis and Characterization of Nanodiamond Reinforced Chitosan for Bone Tissue Engineering. J. Funct. Biomater..

[46] Ma M., Mao Y., Gupta M., Gleason K.K., Rutledge G.C. (2005). Superhydrophobic fabrics produced by electrospinning and chemical vapor deposition. Macromolecules.

[47] Wang X., Yu J., Sun G., Ding B. (2015). Electrospun nanofibrous materials: A versatile medium for effective oil/water separation. Mater. Today.

[48] Li X., Bian F., Lin J., Zeng Y. (2016). Effect of electric field on the morphology and mechanical properties of electrospun fibers. Rsc Adv..

[49] Pacelli S., Acosta F., Chakravarti A.R., Samanta S.G., Whitlow J., Modaresi S., Ahmed R.P.H., Rajasingh J., Paul A. (2017). Nanodiamond-based injectable hydrogel for sustained growth factor release: Preparation, characterization and in vitro analysis. Acta Biomater..

[50] Yao R., Zhang R., Luan J., Lin F. (2012). Alginate and alginate/gelatin microspheres for human adipose-derived stem cell encapsulation and differentiation. Biofabrication.

[51] Chiou B.S., Avena-Bustillos R.J., Shey J., Yee E., Bechtel P.J., Imam S.H., Glenn G.M., Orts W.J. (2006). Rheological and mechanical properties of cross-linked fish gelatins. Polymer.

[52] Liu K.K., Cheng C.L., Chang C.C., Chao J.I. (2007). Biocompatible and detectable carboxylated nanodiamond on human cell. Nanotechnology.

[53] Pacelli S., Maloney R., Chakravarti A.R., Whitlow J., Basu S., Modaresi S., Paul A. (2018). Controlling adult stem cell behavior using nanodiamond-reinforced hydrogel: Implication in bone regeneration therapy. Sci. Rep..

[54] Guarino V., Alvarez-Perez M., Cirillo V., Ambrosio L. (2011). hMSC interaction with PCL and PCL/gelatin platforms: A comparative study on films and electrospun membranes. J. Bioact. Compat. Polym..

[55] Pereira F.A.S., Salles G.N., Rodrigues B.V.M., Marciano F.R., Pacheco-Soares C., Lobo A.O. (2016). Diamond nanoparticles into poly (lactic acid) electrospun fibers: Cytocompatible and bioactive scaffolds with enhanced wettability and cell adhesion. Mater. Lett..

[56] Hopper A.P., Dugan J.M., Gill A.A., Fox O.J.L., May P.W., Haycock J.W., Claeyssens F. (2014). Amine functionalized nanodiamond promotes cellular adhesion, proliferation and neurite outgrowth. Biomed. Mater..

[57] Tsai C.Y., Lin C.L., Cheng N.C., Yu J. (2017). Effects of nano-grooved gelatin films on neural induction of human adipose-derived stem cells. Rsc Adv..

